# RM 8111: Development of a Prototype Linewidth Standard

**DOI:** 10.6028/jres.111.016

**Published:** 2006-06-01

**Authors:** Michael W. Cresswell, William F. Guthrie, Ronald G. Dixson, Richard A. Allen, Christine E. Murabito, J. V. Martinez De Pinillos

**Affiliations:** National Institute of Standards and Technology, Gaithersburg, MD 20899

**Keywords:** AFM, calibration, CD, dimensional standards, HRTEM, lattice-plane selective etch, linewidth, metrology, SCCDRM, reference materials, single-crystal silicon, traceability, uncertainty

## Abstract

Staffs of the Semiconductor Electronics Division, the Information Technology Laboratory, and the Precision Engineering Laboratory at NIST, have developed a new generation of prototype Single-Crystal CD (Critical Dimension) Reference (SCCDRM) Materials with the designation RM 8111. Their intended use is calibrating metrology instruments that are used in semiconductor manufacturing. Each reference material is configured as a 10 mm × 11 mm silicon test-structure chip that is mounted in a 200 mm silicon carrier wafer. The fabrication of both the chip and the carrier wafer uses the type of lattice-plane-selective etching that is commonly employed in the fabrication of micro electro-mechanical systems devices. The certified CDs of the reference features are determined from Atomic Force Microscope (AFM) measurements that are referenced to high-resolution transmission-electron microscopy images that reveal the cross-section counts of lattice planes having a pitch whose value is traceable to the SI meter.

## 1. Introduction

Staffs of the Semiconductor Electronics, the Statistical Engineering, and the Precision Engineering Divisions at NIST, in collaboration with SEMATECH of Austin, Texas, VLSI Standards, Inc., of San Jose, California, and Accurel Systems International, Inc., of Sunnyvale, California, have developed a new generation of prototype Single-Crystal Critical-Dimension Reference Materials (SCCDRM) for calibrating Critical Dimension (CD) metrology instruments that are used in semiconductor manufacturing. Each of the reference materials, which have the NIST designation RM 8111, is configured as a 10 mm × 11 mm silicon test-structure chip that is mounted in a 200 mm silicon carrier-wafer. The fabrication of both the chip itself and the carrier wafer uses the type of lattice-plane-selective etching that is commonly employed in MEMS fabrication. The calibrated CDs of the reference features are determined from Atomic Force Microscopy (AFM) CD measurements that are referenced to high-resolution transmission-electron microscopy images that reveal the cross-section counts of lattice planes having a pitch that is traceable to the SI meter.

## 2. Overview

### 2.1 Goals, Objectives, and Technical Approach

The central goal of this project was to fabricate, calibrate, document, and deliver to SEMATECH and its member companies a selection of CD reference materials with calibrated values as low as 70 nm and having designated reference features with an expanded (i.e., *k* = 2 or 2-sigma) CD uncertainty less than ± 3 nm.

The primary and transfer metrologies that were designated to reach the goal are lattice plane counts as revealed by high-resolution transmission electron microscopy (HRTEM) and Atomic Force Microscopy (AFM) imaging, respectively. This approach to CD calibration has not been implemented previously, as far as we know.

### 2.2 Previous Work

NIST made a prior delivery of CD reference materials to SEMATECH Member Companies that were configured as test chips, each having a single designated reference feature, in 2001.^1^ Their calibrated CD values were in the range 80 nm to 150 nm and had expanded uncertainties of approximately ± 15 nm [1].

The current delivery is also test-chip based with each chip having up to six designated reference features with drawn CDs staggered by 30 nm, each with a stated calibrated CD value and an expanded uncertainty value. For the current 2004 delivery, AFM replaces electrical CD (ECD), which was used in the 2001 delivery, as the transfer metrology.

The calibrated designated reference features are incorporated in a uniquely identified HRTEM target on each distribution chip that has been delivered to respective SEMATECH Member Companies, along with a data sheet listing the CDs and expanded uncertainties of those features. An example of one of these data sheets is shown in Appendix A. A formatted summary of the data shown there is also shown here in Table 1.

The current batch of reference materials includes reference features with calibrated CD values as low as 43 nm and having expanded uncertainties as low as ±1.24 nm. An example of the analysis of the contributions to the expanded uncertainty of a typical feature is shown in Table 2.

## 3. Terminology

The terminology listed below has developed during the course of this work and is used within this report.

SCCDRM: a chip that has been diced from a SIMOX (Separation by Implantation with Oxygen) wafer having special orientation of the principal axes of its test structures, which are patterned into its active device layer, with respect to the silicon lattice to assure near-atomic-scale flatness of replicated silicon features. The CDs of one or more of these monocrystalline features may be calibrated.

AFM Chip: an SCCDRM that has undergone AFM measurement of the CDs of any sub-set of its test-structure features. AFM chips either have been selected as candidates for AFM metrology on the basis of SEM inspection, which is routinely performed for this purpose, or have completed AFM metrology.

Distribution Chip: is an SCCDRM chip that has been set aside for distribution to SEMATECH Member Companies at the conclusion of this project. It has one or more designated reference features, the CDs of which have been calibrated.

HRTEM Chip: an AFM chip which, on the basis of its AFM measurements, has either been selected as a suitable candidate for CD metrology by HRTEM imaging or has completed HRTEM imaging. In effect, AFM imaging is employed to select features and chips for HRTEM imaging. Note that, after HRTEM imaging, an AFM chip can no longer serve as an SCCDRM distribution chip because HRTEM imaging is destructive.

HRTEM Target: a test structure having a geometry that has been designed specifically to facilitate HRPTEM imaging of six reference features with a single FIB (focussed ion beam) cut.

Designated Reference Feature: this is a particular individual feature of an HRTEM target on an HRTEM or AFM chip. The designation encodes the chip and locations where the respective feature may be found by the user.

HRTEM CD: the CD value extracted from an HRTEM image of a designated reference feature of an HRTEM chip. It applies to the entire width of the imaged feature, which includes the thicknesses of the native oxide films on sidewalls.

Apparent AFM CD: the CD of a designated reference feature of an AFM or HRTEM chip, as measured by the AFM.

Calibration Curve: a statistical model that relates the apparent AFM CDs of the HRTEM chips to the corresponding HRTEM CDs.

Calibrated AFM CD: the SI-traceable value of the CD of a reference feature on a distribution chip that is obtained when the apparent AFM CD is referenced to the calibration curve.

## 4. Background and Reference-Material Architecture

### 4.1 Reference-Material Implementation as a SIMOX Test Chip

The reference features were configured as a test chip that is replicated in the device layer of a 150 mm (110) SIMOX wafer [2]. The nominal heights of all the reference features are 150 nm. The device layer is electrically isolated from the remaining thickness of the substrate by a 390 nm thick buried oxide created by oxygen implantation.

**Fig. 1:**
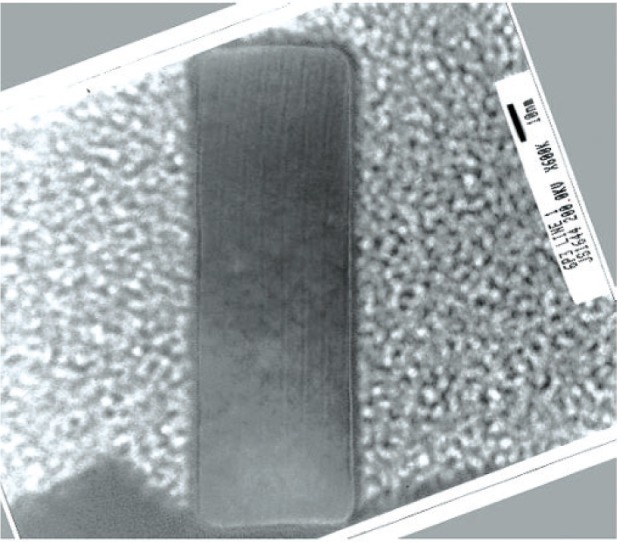
HRTEM of the cross section of reference feature K145 A7 T1 6P3-1. (This nomenclature is described in Sec. 4.3.) The <112> lattice vector is normal to the plane of the paper. The width of this feature is approximately 40 nm. See Fig. 9 for lattice-plane details.

Fabrication begins with the growth of a 10 nm thick oxide film to serve as a hard-mask material. The test-chip image to be described in Sec. 4.2 below is then projected into resist so that its principal axes are orientated to a <112> lattice direction. The latter is established by transferring of a special-purpose angular fiducial pattern to the hard mask and transferring it to the silicon with a deep, lattice-plane-selective etch. Lattice orientation is subsequently determined from visual inspection of the features of the pattern. The reference-material test chip pattern is then photo-lithographically transferred to the hard mask at the correct orientation to the lattice, as revealed by the features of the etched angular fiducial pattern. It is then replicated in the p-type silicon surface layer of the substrate by lattice-plane selective etching. Tetra-methyl-ammonium hydroxide (TMAH) etches (111) silicon-lattice planes at a rate 10 to 50 times more slowly than it etches other planes, such as the (110) surface of the wafer, allowing the (111) planes of the reference-feature sidewalls to behave as lateral etch stops. Aligning reference-features with <112> lattice vectors in the (110) surface of the wafer results in their having planar, vertical, (111) sidewalls. An actual reference-feature cross section is illustrated in the HRTEM image shown in Fig. 1.

### 4.2 Chip Layout and Feature-Identification Scheme

**Fig. 2:**
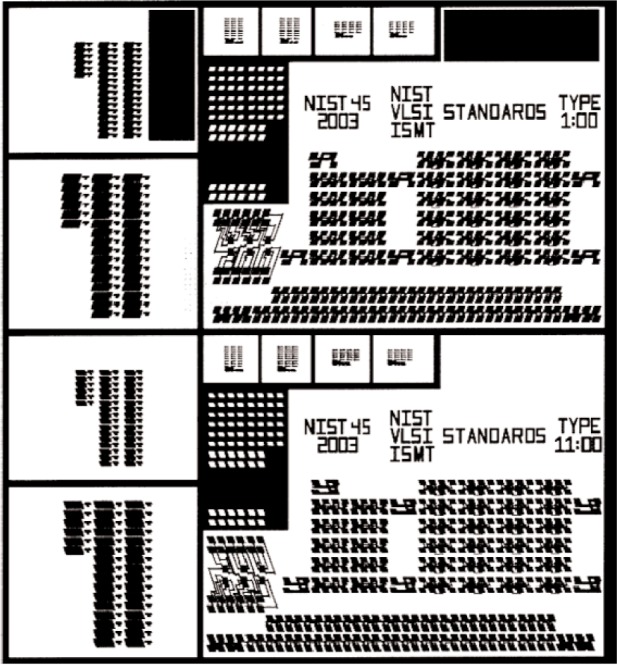
Layout of the 10 mm by 11 mm NIST45 test chip.

**Fig. 3:**
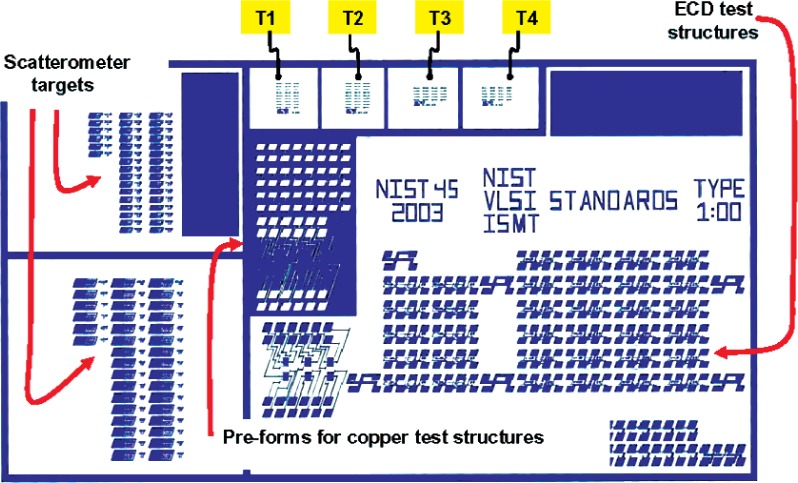
The upper (1 o’clock) section of the NIST45 SCCDRM chip layout has, among other structures, the HRTEM target arrays T1 through T4.

Figure 2 shows the layout of the 10 mm × 11 mm NIST45 test chip. The principal axes of the test-structure geometries in the upper and lower sections of the test-chip layout are drawn to be oriented to the <112> and <−112> directions. Figure 3 shows the upper (so-called, 1 o’clock) section. It identifies, among other groups of test structures, the HRTEM-target arrays T1 through T4 where HRTEM reference features are located when they are on the 1 o’clock section of the test-chip layout. Note that the corresponding HRTEM-target arrays in the lower section of the test-chip layout, which can be seen from inspection of Fig. 2, are identified as B1 through B4. Of the 10 distribution chips that have been delivered to SEMATECH, some have CD-calibrated designated reference features in the T1-T4 arrays and some have them in the B1-B4 arrays.

### 4.3 Calibrated Reference-Feature Identification Scheme

Individual features are identified with a designation which uniquely identifies each respective feature according to its:

<Process Job>,
<Chip Number>,
<Target-Array Number>,
<Target Number>, and its
<Feature Number>.

**Fig. 4:**
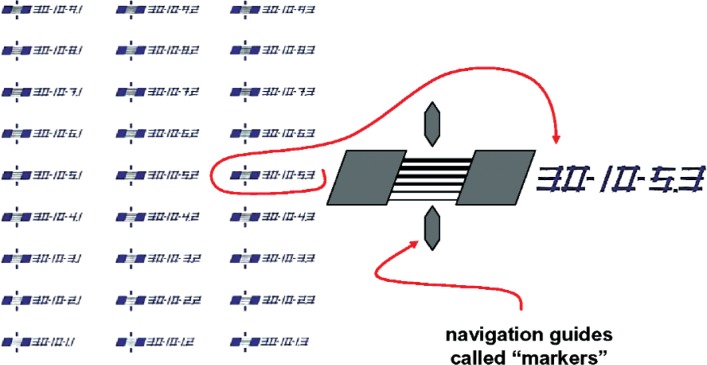
One of the HRTEM target arrays, the one labeled T1 in Fig. 3, in more detail. HRTEM Target # 30-10-5.3 has been enlarged to show the six-feature architecture that is common to all targets. Note that the annotation 30-10, appears on targets located in both the T1 and B1 target arrays.

For example, K145-A10-T3-5p3-F4, is a reference feature from the K145 process job, on chip A10, in the T3 HRTEM target array, specifically the 5p3 target, where it is the fourth feature. The process job number is chosen as a laboratory-notebook page-number reference and is also archived as an electronic document at NIST. The designation-component “5p3” corresponds to target “5.3” in Fig. 4, where the lettering “5.3” is actually patterned into the substrate, but could not be incorporated into a file name.

### 4.4 HRTEM-Target Architecture and Target Locations

**Fig. 5:**
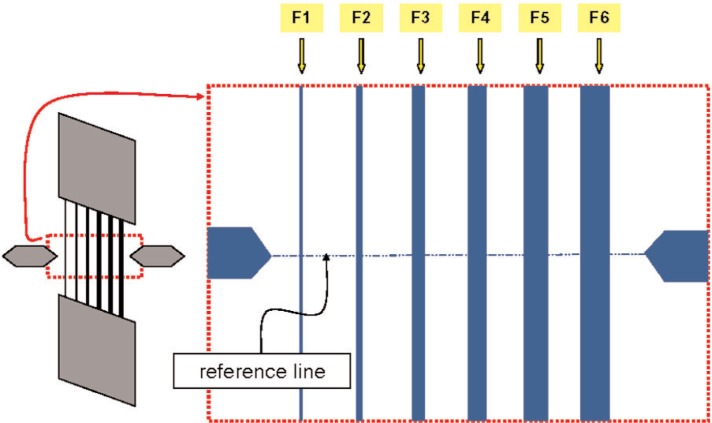
A schematic drawing of the target, rotated counter-clockwise by 90 degrees relative to the orientation shown in Fig. 4. The labels F1 through F6 identify individual features of the target. F1 is always the narrowest, as-drawn feature.

Figure 4 shows one of the HRTEM target arrays, the one labeled T1 in Fig. 3, in more detail. In this figure, HRTEM Target # 30-10-5.3 has been enlarged to show the six-feature architecture that is common to all targets.^2^ The reference-features are designed so that the drawn linewidths increase progressively from the lower part to the upper part of each target and from the lower left to the upper right of each HRTEM target array. Note that the individual reference features in Fig. 5 are identified as F1 through F6 from left to right.

## 5. Screening and Chip Selection for AFM

As indicated in Sec. 2.1, the primary metrology that has been selected to reach the project’s goals is counting of lattice planes that are illuminated by HRTEM phase-contrast imaging [3]. This technique, however, cannot be used for the reference materials that are to be delivered to an end-user because it is destructive. It is also very expensive, a fact that becomes clear from the descriptions provided later in Secs. 7.1 and 7.2. Therefore, a benign non-destructive metrology, in this case AFM, is used for transfer metrology. However, AFM has relatively low throughput and higher cost than SEM (Scanning Electron Microscopy). Accordingly, SEM pre-screening was implemented for this project to facilitate selection of as-fabricated chips for AFM metrology, in rather the same way that AFM metrology was in turn applied to the judicious selection of AFM chips for HRTEM imaging. The necessity for pre-screening resulted from the fact that the special silicon-substrate and post-processing that was employed to fabricate the reference materials is prone to impart local, randomly located, structural defects in the reference features. This may be partly due to the fact that fabrication was not performed in a clean room. In any case, it was possible to compensate for the defects by careful SEM inspection after patterning, and before AFM inspection, and ranking of candidate SCCDRM chips. In general, the optical and SEM inspections sought to identify HRTEM targets whose features visually appeared to be CD-uniform, unbroken, and free from contamination and other defects, and had at least one feature with a sub-100 nm CD. Each of the candidate chips had multiple test-structure instances, each with a number of candidate features, as has been shown in Fig. 4 and Fig. 5.

High-resolution top-down SEM imaging at 15 kX magnification and 5 keV to 10 keV was implemented for pre-AFM inspection. Three features of each target were captured in each of two successive images. A montage of a pair of these images, which are typical of those that were acquired, is shown later in Fig. 10. Since several hundred chips were selected for SEM inspection by systematic high-resolution optical inspection, it was necessary to implement a database to archive the large number of images to facilitate selection of AFM chips ostensibly having more preferable reference-feature properties. This database facility further enabled an enhancement to the selection process through highly systematized SEM-image processing that characterized each candidate reference feature, HRTEM target, and SCCDRM chip [4]. These parameterizations then allowed automated interrogation of the database, which resulted in the benefits of rapid identification of the “best” SCCDRM chips for AFM metrology in quasi-real time. A further refinement to the database then allowed archiving the silicon-processing conditions and, at a later date, the AFM and HRTEM measurement data that were extracted from chips that had been selected for these more costly measurements. It is anticipated that this database embeds a wealth of information that could, in principle, be extracted to optimize the overall reference-material fabrication and calibration processing in terms of generating narrower CDs having still lower uncertainties.

One disadvantage of using SEM for pre-inspection was its well-known tendency to deposit hydrocarbon contamination on the features, which challenged the calibration procedures [5, 6]. This contamination increases the apparent linewidth that is measured by the AFM. In addition, some forms of residual contamination, including moisture, may adversely affect the imaging stability of the CD-AFM tip–resulting in so-called “tip skipping” during the scan. Generally, these issues were resolved by use of a cleaning process prior to AFM imaging. This process, which was developed for this project, was not fully optimized but was observed to be quite effective. Basically, for each batch of chips, the cleaning process involved ultrasonic cleaning of two sets of quartz-ware in high-purity, laboratory-grade, isopropyl alcohol (IPA).^3^ All quartz-ware was subsequently baked in a vacuum oven at 200 ºC for 1 h. The SCCDRM chips are then flat-rinsed in running DI (deionized) water, blow-dried in clean nitrogen, and immersed in IPA, which is located in one of the pre-cleaned sets of quartz-ware. They are then removed from the IPA, again blow-dried, and then transferred into the other set of quartz-ware which is then returned to the vacuum oven for several hours. Note that the quartz-ware and chips should be thoroughly air-dried before placing them in the oven, as IPA/air mixtures can be explosive.

This procedure was generally successful in preventing “skipping” of the CD-AFM boot-tip probe, which was essential for AFM imaging. However, it appears that further development of this cleaning process would be advantageous as far as totally removing the SEM-induced hydrocarbon contamination and/or all other residues that are sometimes left on the reference-features’ surfaces after patterning. Plasma cleaning is one possible approach that has been reported [6].

## 6. AFM Metrology

### 6.1 Veeco Dimension X3D Critical Dimension Atomic Force Microscope

The AFM instrument used in this work is the Veeco Dimension X3D Model 340 (X3D).^4^ This tool is installed in the Advanced Technology Development Facility at SEMATECH, and it has been implemented as a Reference Measurement System (RMS) [7].

The unique aspects of CD-AFM operation are that force sensing occurs along two axes (one vertical and one lateral) and that flared or “boot-shaped” tips are used. This allows imaging of near-vertical sidewalls, which is not possible with the conical probes used in a conventional atomic force microscope. Both conventional atomic force microscopes and CD atomic force microscopes are sensitive primarily to the topography of the surface and exhibit very little dependence on material composition. As such, CD-AFM is an ideal choice for transfer metrology for the subject reference-material features.

### 6.2 Extraction of Apparent AFM Values From Measurement Data

**Fig. 6:**
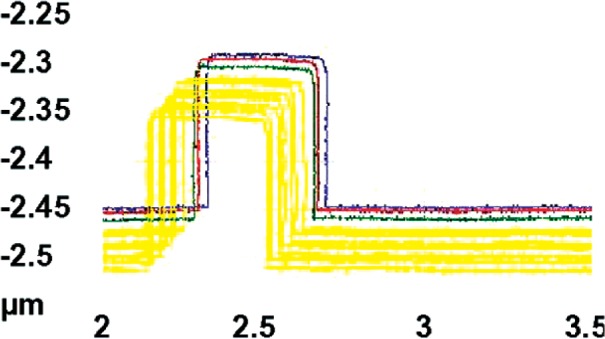
Example of CD-AFM profiles extracted from an HRTEM-target feature. Three are highlighted here. Only a portion of the image is displayed so that more detail is visible.

The markers on the HRTEM targets, which have been shown in Fig. 4, Fig. 5, and later in Fig. 10, were used for navigation. In cases where the reference line of Fig. 10 was not exactly perpendicular to the reference features, each AFM image was referenced to the more left of the two markers. The length and width of each as-captured AFM image included measurements of all six features of the target and its width extended for a total of 2 µm centered approximately on the intersection with the reference line drawn from the left marker pointer. The spacing between adjacent AFM line-scans in the images was 25 nm. A typical AFM measurement profile for one feature consisted of approximately 78 line-scans. An example of a typical set of line-scans extracted from an AFM image is shown in Fig. 6.

**Fig. 7:**
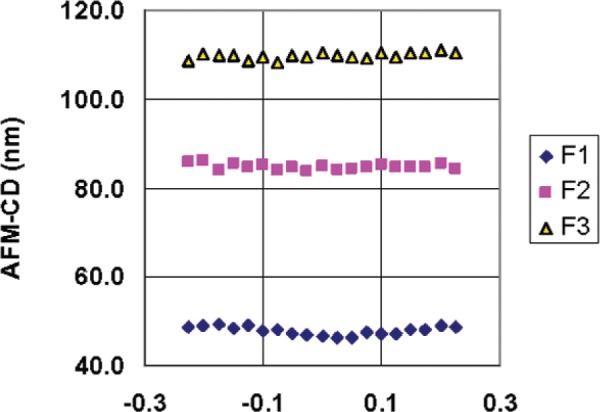
An example of the AFM measurements for features F1, F2, and F3 from HRTEM chip K147-D1. The x-axis (location) values are centered on the reference line that has been shown in Figure 5 and extend 0.25 µm in each direction from the location corresponding to 0.0 µm.

The linewidth analysis was performed with the Veeco Nanoscope v6.22r1 software currently supplied with the Dimension X3D. Each of the features in the images was individually windowed and analyzed sequentially. For purposes of this calibration, the width was calculated at the half-height of each feature at a series of locations (i.e., scan line numbers) along the features. An example of these measurements for features F1, F2, and F3 from HRTEM chip K147-D1 is shown in Fig. 7. In this illustration, the x-axis values are centered on the reference line that has been shown in Fig. 5 and extend 0.25 µm in each direction.

**Fig. 8:**
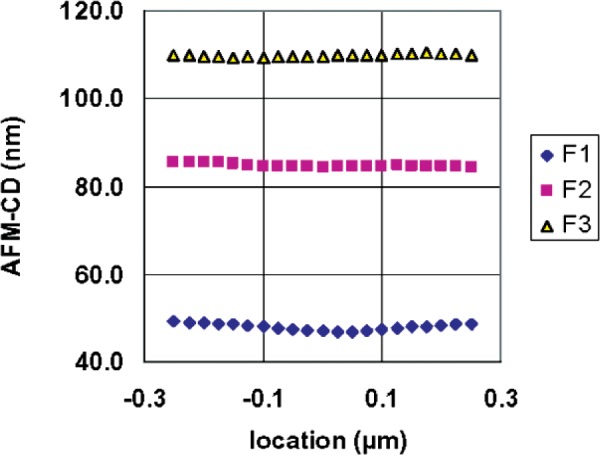
The results illustrated here provide a visual indication of typical levels of width uniformity of the central segments of different features in the same HRTEM target and, by comparison with those in Fig. 7, the impact of the 7-point moving-average smoothing.

Measurements similar to those shown in Fig. 7 were recorded for all six features of one or more designated targets on each of a set of 23 chips. Four of these were selected for HRTEM, and the remainder were set aside as distribution chips. The measurements were then further processed for calibration-curve construction in the case of the HRTEM chips, or for determination of the CD values in the case of the distribution chips. As part of the analysis, the “raw” AFM measurements were smoothed with an equally weighted 7-point moving average to reduce the effects of measurement noise. The results of applying the moving average model to the raw measurements that are shown in Fig. 7 are shown in Fig. 8. The results illustrated in Fig. 7 and Fig. 8 graphically indicate typical levels of CD uniformity of different features in the same target, and the impact of smoothing by taking 7-point moving averages.

The raw AFM measurements were processed differently for the HRTEM chips and the distribution chips. In the case of the HRTEM chips, AFM data centered around a 0.5 µm feature-segment length where the HRTEM measurements were taken were used to compute the apparent AFM CD. Thus, the apparent AFM CD that was used for each calibration-curve point included data from 21 adjacent line scans. In the case of the distribution chips, the AFM data were centered at the intersection of the reference line, which was illustrated in Fig. 5, with the respective designated feature. By comparison, the apparent AFM value, which was specified for each feature that was calibrated for distribution, used data from only five adjacent line scans. Further details of the analyses of apparent AFM values from the respective sets of measurement data are provided in Secs. 8.1 and 8.3.

### 6.3 Instrument Calibration

The AFM measurements on all the SCCDRM chips, for both HRTEM and for distribution, were performed using the same procedure [8]. Since the X3D has been implemented as an RMS, its performance and uncertainties have been well characterized. It was critical that the instrument scale calibration and offset (i.e., bias of the apparent width relative to the SI meter) be the same for all of the measurements, because this is an assumption of the analysis discussed in Sec. 8.

The same traceable scale calibration, which has a standard uncertainty of ± 0.1 %, was used for all of the SCCDRM measurements. While the absolute standard uncertainty of the routine AFM tip width calibration that was available at the time of the measurements was ± 5 nm, it was possible to measure relative widths much more accurately. For features with vertical sidewalls and good uniformity, it is possible to measure relative widths with an expanded uncertainty of approximately ± 1 nm. Since tip wear during measurements directly increases the relative uncertainty, it was necessary to perform measurements on a “monitor” specimen before and after every measurement on an SCCDRM chip. The same monitor specimen was used for both the HRTEM chips and the distribution chips. In this manner, it was possible ensure that all the apparent AFM widths, although performed using different tips at different times, were measured using the same relative calibration of tip width. In other words, all of the measured tip widths, and thus the apparent feature widths, shared a common bias relative to the SI meter to within an expanded uncertainty of ± 1 nm.

## 7. HRTEM Imaging

**Fig. 9:**
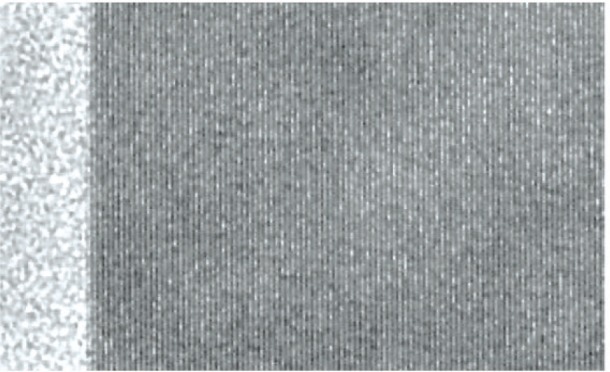
An example of an image of (111) fringes. The (111) lattice planes have a pitch of 0.313 560 156 nm ± 0.000 000 012 nm in units traceable to the SI meter.

HRTEM images of thin cross-section membranes generate phase-contrast fringes that correspond to the (111) lattice planes which constitute the linewidths of the designated features on SCCDRM chips. An example of an image of (111) fringes is shown in Fig. 9. The lattice plane pitch has a pitch that is traceable to the SI meter [9]. The lattice-plane counts revealed by the fringes thus enable tracing the linewidths of the designated features on the HRTEM chips to the SI meter [10].

### 7.1 Platinum Ribbon Deposition

**Fig. 10:**
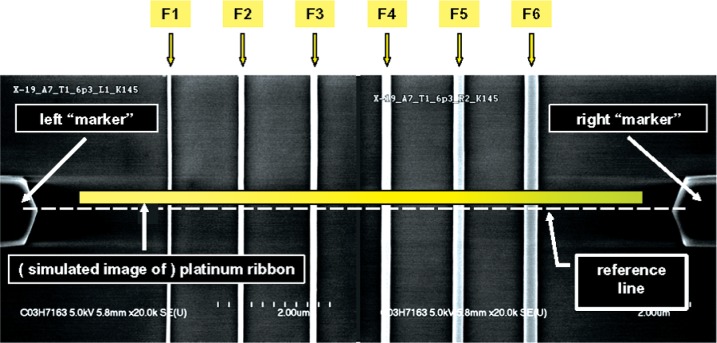
The HRTEM target is placed in a focused ion beam/scanning electron microscope tool to mark the location to be cross-sectioned with an electron-beam-assisted platinum deposition. The length of the resulting platinum ribbon mark is approximately 8 µm.

The designated target of the HRTEM chip is prepared for HRTEM imaging with a process that has been optimized to ensure that the surfaces of the reference features are not damaged [11]. surfaces of the reference feature during the process steps that follow. After coating, the specimen is placed in a focused ion beam/scanning electron microscope (FIB/SEM) tool to mark the location to be cross sectioned with an electron-beam-assisted platinum deposition. The resulting platinum ribbon mark is approximately 0.5 µm by 8 µm, as illustrated in Fig. 10. The platinum ribbon also serves to protect the reference features during the next step, which is deposition of a protective platinum box, 8 µm by 20 µm.

At this point, the specimen is removed from the FIB/SEM and tripod polished to a thickness of 30 µm. The 30 µm thick membrane is then silver mounted on a half grid and returned to the FIB/SEM and thinned. At the beginning of this process, a 30 kV gallium beam is used for rapid thinning; the final thinning uses a 10 kV beam to prevent damage to the reference feature. This thinning process targets the center of the 0.5 µm region defined by the electron-beam-assisted platinum deposition and continues until the reference feature becomes electron transparent, at which point it has a thickness typically between 25 nm and 30 nm.

### 7.2 Extraction of the HRTEM CDs of Designated Features on the HRTEM Chips

In previous work, we reported a task to develop an automated procedure for determining the fringe counts [12]. However, to further reduce the calibrated-AFM CD uncertainties of the distributed-chips’ designated reference features, we have now implemented an expanded manual counting procedure. Specifically, each of four operators independently counted the fringes at three heights in each reference feature. Each operator averaged his or her three linewidth measurements for each feature. In a few cases in which larger than expected disagreements between operators were observed, the operators were directed to repeat their fringe counts. No interaction between the operators during this entire process took place. If asked to recount the fringes on a particular HRTEM image, the operator was not informed whether his or her prior average measurement was higher or lower than the corresponding ones made by the other operators. In each case in which a recount was requested, a counting error was found and, generally, after each requested recount the agreement between operators was less than 1 nm. Since the HRTEM must account for the native silicon dioxide on the sidewalls of the feature, beyond the easily countable fringes produced by the silicon lattice, the operators were asked to use adjacent fringes as a ruler to measure the thickness of the native oxide.

## 8. Calibration

After the HRTEM and the AFM images of the twelve features on the two chips that were selected for HRTEM had been captured, their HRTEM CDs were extracted according to the description in Sec. 7.2 above. The method by which the corresponding AFM CDs were obtained is now described in Sec. 8.1 below. The two sets of measurements are reconciled with the generation of the calibration curves to be described in Sec. 8.2 below.

### 8.1 Extracting Apparent AFM CD Values and Uncertainties for the Calibration Curve

The extraction of AFM CD values from the respective sets of line scans was performed as described in Sec. 6.2 above. Four chips were originally measured by HRTEM, but analysis of the calibration data from the four chips indicated that two of the chips may have been affected by sidewall-surface contamination. These two were subsequently not used in the final calibration analysis. *The possible presence of permanent surface contamination on the distribution chips is not a major concern because the X3D AFM measures the total CD of each feature, which includes contributions from both the crystalline silicon core, and the native oxide films on each of the two sidewalls, as well as any residual contamination.* The following sections describe in more detail the procedure that was unique to analysis of the AFM measurements that were made exclusively on the HRTEM chips.

#### 8.1.1 Apparent AFM CD Values for HRTEM Chips

In order to determine, for each designated feature on the HRTEM chips, an appropriate AFM CD to associate with its HRTEM CD, the location of the imaged cross section membrane relative to the markers on the respective targets was established from inspection of the top-down SEM images. A montage of examples of two of these is shown in Fig. 10. Since the position of each AFM line scan with respect to the reference line between the markers is known, the range of possible locations from which the corresponding HRTEM image was extracted was available. Therefore, for each designated feature on the HRTEM chips, a set of CDs was obtained by averaging the 21 adjacent AFM line scans, smoothed with a 7-point moving average, as discussed in Sec. 6.2, of a 0.5 µm feature segment matching the observed location of the centerline of the platinum ribbon. However, the HRTEM vendor asserted that the HRTEM images were much more likely to represent locations nearer to the centerline of the platinum ribbon. *Therefore, during averaging, a weighting function that weighted each smoothed line-scan value according to the inverse of its distance from the centerline was employed to estimate the appropriate AFM CD to associate with the corresponding HRTEM CD.*

#### 8.1.2 AFM-Value Uncertainties for HRTEM Chips

Uncertainties attributed to each AFM CD, that were obtained according to the preceding paragraph, arose from the random variation observed in the AFM measurements, the reproducibility of the relative tip-width calibration, and the interaction of feature non-uniformity as observed with the AFM and possible errors in navigating to the membrane where the HRTEM measurement was made with the atomic force microscope. *The uncertainties due to the relative tip width calibration and the CD non-uniformity/navigation were treated as Type B uncertainties according to ISO-published methods* [13, 14].*Since it is believed to be more likely that the HRTEM measurements were made nearer to the center of the platinum ribbon rather than to its edges, and that any navigational errors were small, a triangular probability distribution was used to convert an upper bound on the range of possible CDs in the AFM window to a standard uncertainty by dividing the range by 2√ 6.* The standard uncertainty for the AFM CD was then computed by combining the uncertainties of the weighted mean of the AFM measurements, the relative tip width calibration, and the CD non-uniformity/navigation using propagation of uncertainty. In this case, that is calculated by combining the uncertainties by “root-sum-squares.” The combined standard uncertainty of the AFM CD is assumed to have infinite degrees of freedom for several reasons [13, 14].

1. The random uncertainty of the weighted mean of each AFM result is based on a large amount of data (approximately 78 points/feature),
2. The uncertainty from the relative tip width calibration is based on experience across a wide range of AFM measurement applications, and,
3. The uncertainty due to CD non-uniformity/navigation is based on an upper bound over the range of possible CDs.

### 8.2 Calibration-Curve Construction and Statistics

**Fig. 11:**
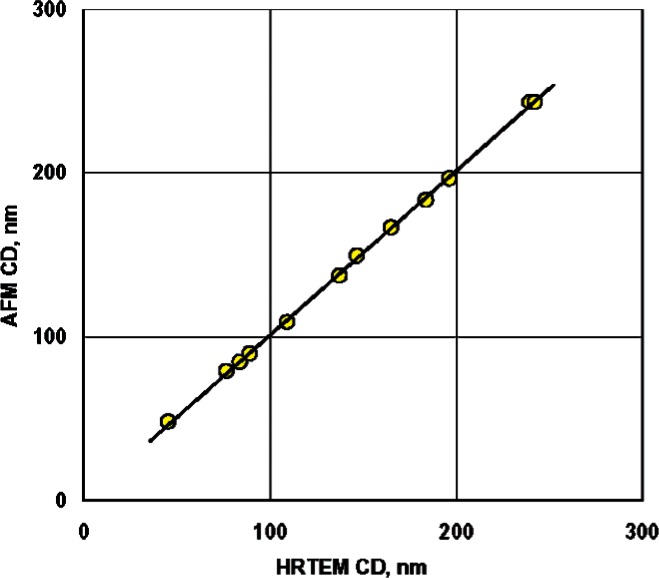
The apparent AFM CD values for HRTEM chips and their corresponding HRTEM CD values with a straight-line calibration curve obtained with weighted least-squares fitting.

The apparent AFM CD values for HRTEM chips, obtained as described in Sec. 8.1.1, and their corresponding HRTEM CD values, obtained as described in Sec. 7.2, are shown in Fig. 11. Because of variations in the uniformity of the AFM CDs of the features, the initial calibration model,

$$CD_{\text{AFM}}=\beta_{0}+\beta_{1}\cdot CD_{\text{HRTEM}}$$

where *β*_0_ is the intercept of the regression line and *β*_1_ is its slope, was fitted by weighted least-squares regression with weights inversely proportional to the variances of the respective AFM CD values. The latter were obtained according to the descriptions in Sec. 8.1.2. These weights, although estimated individually, should be reasonably stable since each weight is based primarily on an estimate from a bootstrap re-sampling procedure with approximately 78 data points per feature [15, 16]. The calibration curve from the fit of the regression model is also shown in Fig. 11. The numeric output is shown in Table 3.

The fact that the slope of the linear calibration, 0.996 +/− 0.012,^5^ does not differ significantly from unity indicates that the independent traceable scale calibration of the AFM agrees with the HRTEM results, as expected prior to the analysis of the measurements. Because of this, and to take into account the uncertainty in the HRTEM CD measurements directly, a slope of unity was assumed and a simpler “offset-only” model was used to estimate the difference between the apparent AFM CDs and the HRTEM CDs. Physically, this offset corresponds to the bias in the CD-AFM tip width calibration that was used when the data were acquired.

To estimate the offset of the atomic force microscope, a weighted average of the difference between each apparent AFM CD and the corresponding HRTEM CD was used. The weights were inversely proportional to the square of the combined standard uncertainty for each difference. Using individually estimated weights is usually problematic because one of the key assumptions underlying the use of weighted averages is that the weights are known without error and there are usually not enough data to justify the estimation of an individual weight for each data point. In this case, however, since the minimum number of effective degrees of freedom over all sources of uncertainty for each difference is 66 degrees of freedom (from the pooled estimate of the HRTEM CD uncertainty) the assumption that the weights are known without error is quite reasonable.

**Fig. 12:**
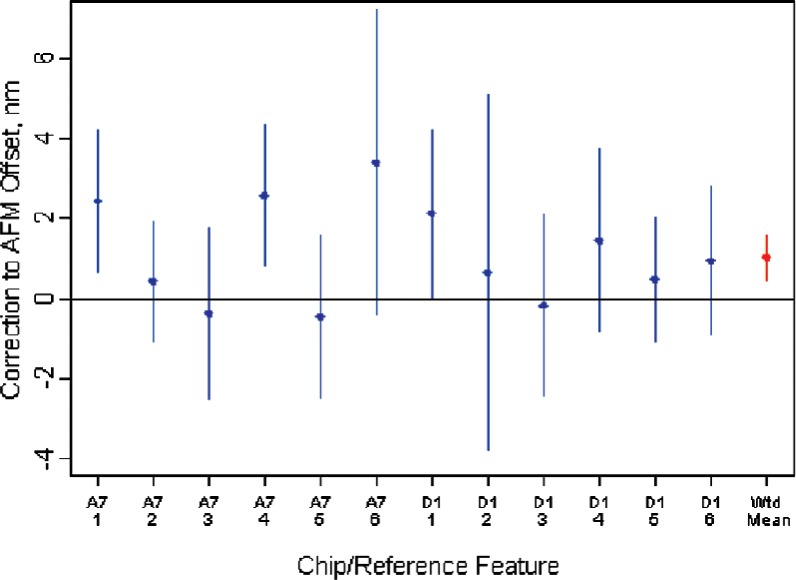
Comparison of the individually estimated AFM offsets and the weighted-mean offset. The uncertainties shown in the plot are the expanded uncertainties (*k* = 2).

Use of the offset-only model and the assumption of individual known weights provided an estimated offset of the AFM of 1.03 nm, this offset having a combined standard uncertainty of ± 0.29 nm. The standard uncertainty of the estimated AFM offset includes the uncertainty of each apparent AFM CD and the standard uncertainty of the associated HRTEM CD. A plot that compares the individually estimated AFM offsets and the weighted-mean offset is shown in Fig. 12. The uncertainties shown in the figure are expanded uncertainties (*k* = 2).

### 8.3 Distribution-Chip CD and Uncertainty Distributions

For each designated feature of the distribution chips, calibrated AFM CD values are determined by subtracting the AFM offset from the apparent AFM CDs for each feature. The uncertainty of each calibrated CD is estimated using propagation of uncertainty, which reduces to summing the uncertainties by “root-sum-of-squares” in this case. The combined uncertainties of the calibrated AFM CDs include the respective uncertainties in the apparent AFM CD, as discussed in Sec. 8.1.2, and the uncertainty of the estimated AFM offset, as described in Sec. 8.2.

**Fig. 13:**
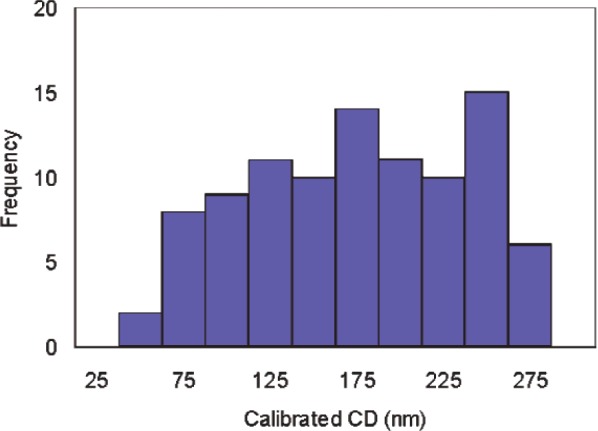
Distribution of distribution-chip calibrated CD values.

**Fig. 14:**
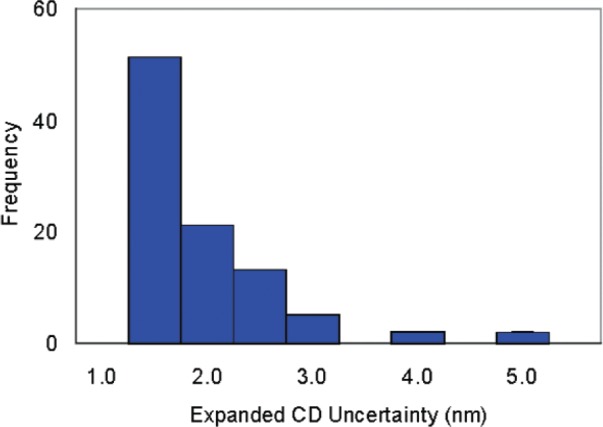
The distribution of distributed-chip CD uncertainties.

Figure 13 and Fig. 14 provide an overview of the calibrated AFM CDs of the distribution chips and their expanded uncertainties. In Fig. 13 and Fig. 14, the breadth of the distribution results from the fact that the plot depicts results extracted from six features from each of multiple targets. The content of the data attachment that would have accompanied the delivery of the distribution chip K153-HH is shown as an example in Table 4. The data attachment itself is reproduced in Appendix A. Table 5 shows an example of the uncertainty budget for feature K153-HH-T1-7p3-F1.

## 9. Carrier-Wafer Implementation

**Fig. 15:**
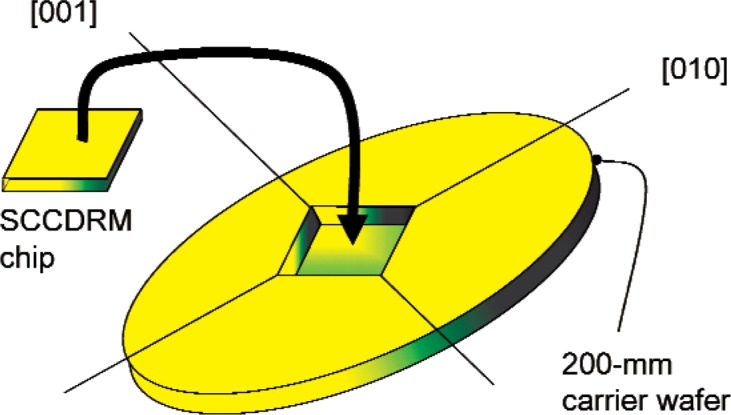
The 200 mm carrier wafer designed to accommodate an SCCDRM chip having dimensions of approximately 10 mm by 11 mm.

Figure 15 illustrates a 200 mm carrier designed to accommodate an SCCDRM chip having dimensions of about, in this case, 10 mm by 11 mm. The selection of the carrier-wafer lattice directions as shown in Fig. 15 assures exact rectangularity of the recessed pocket, which accommodates the SCCDRM, flatness of the pocket floor, control of lateral dimensions of the pocket to within several microns, and having pocket sidewall slopes of 54.37°. This value is crystallographically defined.

**Fig. 16:**
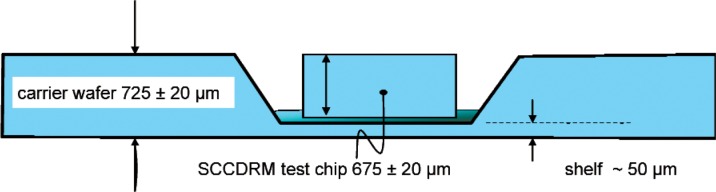
A schematic cross section through the SCCDRM test-chip pocket in the carrier wafer.

In addition, the photo-lithography that generates the pocket’s features can readily produce any desired pattern of reference marks with sub-micrometer placement accuracy that is unobtainable by any other known means [17]. Figure 16 shows a cross section through the SCCDRM test-chip pocket in the carrier wafer. This example applies to the installation of SCCDRM chips into carrier wafers that are micro-machined from standard 200 mm wafers having thicknesses of 725 µm. The SCCDRM chips were diced from 150 mm wafers, which have a standard thickness of 675 µm.

The carrier wafers were fabricated by variations of silicon micro-machining techniques. For the purposes of mounting the distribution chips, the 200 mm (100) starting wafers were first oxidized to provide an in-situ hard-mask material for TMAH lattice-plane selective etching. Photolithography of one side of the wafer was conducted to replicate the SCCDRM-chip pocket at its desired location. The next step was selective removal of the hard-mask oxide by 17 % buffered oxide etch solution. The pockets were then generated with extended lattice-plane-selective TMAH etching.

Co-planarity of the upper surfaces of the reference-artifact test-chip and the carrier wafer was achieved by careful application of an optical flat after adhesive was applied between the lower surface of the SCCDRM chip and the floor of the recessed pocket.

## 10. Recommendations for Use of the SCCDRM

To use one of the designated SCCDRM features, the user must determine the CD at the center of the feature as indicated by the built-in reference markers shown in Fig. 4. This could be done by scanning along the line for some distance (say 0.25 µm) around the center of the feature and averaging the results or by fitting an appropriate model to the linewidth results by scan line and predicting the CD at the location aligned with the marker. The measurements of the SCCDRM features should be made near the middle of the line height, where the AFM calibration data were taken. After the user has determined the CD of the reference feature, the offset for the measurements of new specimens can be obtained by comparing the result obtained from measuring the reference feature with its calibrated value. When a new specimen is measured, that offset can be used to correct the new measurement result to the traceable value. Note that the method used to measure the new samples should be as similar as possible as the method used to measure the reference feature on the SCCDRM. Any differences in the measurement procedures may preclude establishing traceability. The overall uncertainty of the measurement of a new specimen depends on uncertainties from several sources that include the user’s measurement of the RM8111 reference feature and the uncertainty in its calibrated value. All such sources must all be accounted for in the reported uncertainty for the CD of the new specimen.

If the SCCDRM is being used only to evaluate the AFM tip width calibration offset (or bias), then the AFM scale should be independently calibrated using a traceable pitch standard prior to measuring the SCCDRM. In principle, the SCCDRM could then be used directly for traceable tip width calibration by subtracting the calibrated width value from the raw apparent width (i.e., the width measured using no tip correction). Subsequent width measurements are then traceable. Typically, however, users may find it more practical to use the SCCDRM to determine the bias of their existing tip width calibration specimen. In this case, the user should measure the SCCDRM using his or her current tip width calibration procedure. The difference between this apparent width and the calibrated value gives a measure of the bias in the user’s existing tip calibration. This offset should then be used to correct the assumed width value of the user’s tip calibration standard to a traceable value. Subsequent tip width calibrations are then traceable. Because there is an additional measurement step, this would generally result in slightly larger uncertainties than direct tip width calibration using the SCCDRM. However, this contribution may not be significant, and the convenience of this approach may outweigh other considerations.

To establish uncertainty of width measurements on their own specimens, users should consider all the relevant sources of uncertainty. These sources should include: (1) the uncertainty in the calibrated value of the reference feature obtained from the data attachment accompanying this report, (2) the statistical (type A) uncertainty in the user’s measurement of the reference feature, (3) the statistical uncertainty in the user’s measurement of the new specimen, (4) the statistical uncertainty of the user’s routine tip calibration (unless the SCCDRM is being used directly for this), and (5) the uncertainty of the scale calibration. In addition, there may be other sources unique to the user’s circumstances and application that should be considered.

Evaluation of the AFM tip-calibration offset can be performed using results on only a single SCCDRM feature. However, if more features are measured, the additional information can be used to advantage. The most apparent possibility is to use the results from multiple features to obtain a more accurate estimate of the bias. However, depending upon the accuracy of the user’s existing scale calibration, it might also be useful to use results on multiple features as a check on scale calibration and linearity. As a final caveat, please note that the reported values represented the CDs at the time of measurement in April 2004. Although we fully expect the CDs to remain stable over time, measurements of a selection of SCCDRM CDs will continue to be monitored by NIST. If changes are observed, this information will be reported to all known users of these SCCDRMs.

## Figures and Tables

**Table 1:** The CDs and expanded uncertainties for an actual distribution chip typical of those delivered to an SEMATECH Member Company. The complete Data Attachment is shown in Appendix A

Feature	CD (nm)	Expanded uncertainty (nm)
F1	70.96	1.42
F2	71.55	1.57
F3	115.17	1.74
F4	136.08	1.47
F5	175.95	1.25
F6	231.73	1.58

**Table 2:** Analysis of the contributions to the expanded uncertainty for Feature F1 of the Distribution Chip for which the results are shown in Table 1

Source of uncertainty	Uncertainty estimate (nm)
Random Measurement Variation—AFM	0.23
AFM Reproducibility	0.50
CD Non-Uniformity/Navigation	0.34
Estimated AFM Offset	0.29
Combined Standard Uncertainty	0.71
Expanded Uncertainty (*k* = 2)	1.42

**Table 3:** The statistics of the regression that generated the calibration curve in Fig. 11

Coefficient standard	Value	Standarduncertainty	Degrees of freedom
Intercept^a^	1.5519 nm	0.8000	10
slope^a^	0.9960	0.0053	10

Residual standard Error	1.4960 nm with 10 degrees of freedom		
Multiple *R*-squared	0.9997		

a Note: the uncertainties of the intercept and slope are both based on the residual standard deviation.

**Table 4:** The content of the data attachment that would have accompanied the delivery of distribution chip K153-HH

Feature	CD (mm)	Expanded uncertainty (nm)
F1	70.96	1.42
F2	71.55	1.57
F3	115.17	1.74
F4	136.08	1.47
F5	175.95	1.25
F6	231.73	1.58

**Table 5:** Analysis of the contributions to the expanded uncertainty for feature K153-HH-T1-7p3-F1

Source of uncertainty	Uncertainty estimate (nm)
Random meas. variation—AFM	0.23
AFM reproducibility	0.50
CD non-uniformity/navigation	0.34
Estimated AFM offset	0.29
Combined standard uncertainty	0.71
Expanded uncertainty (*k* = 2)	1.42

## Footnotes

1 A glossary of the terms used in this section and in the rest of this document is provided in Sec. 3 “Terminology.”

2 The annotation 30-10, which is shown in Fig. 4, indicates that the target is located in either the T1 or in the B1 target-array. The other targets have similar but different annotations that correspond to the target arrays in which they are located.

3 The practitioner is advised not to locate IPA in the ultrasonic tank directly but to fill the tank with water, for example. Another vessel containing the IPA can then be partially submerged in the water in the tank. Since this does not completely prevent generation of IPA vapor, adequate venting of the workspace is highly recommended.

4 Certain commercial equipment, instruments, or materials are identified in this document in order to specify adequate measurement procedures. Such identification does not imply recommendation or endorsement by the National Institute of Standards and Technology, nor does it imply that the materials or equipment identified are necessarily the best available for the purpose.

5 The estimate of the expanded uncertainty of the slope is computed by multiplying the standard uncertainty of the slope (0.0053) by a coverage factor of *k* = 2.228 obtained from the Student’s t-distribution with 10 degrees of freedom. This may be an under-estimate because the uncertainty in the HRTEM CDs may not be fully accounted for in parameter estimates of the linear regression. Since the 95 % confidence interval for the slope includes the value 1 with this smaller estimate of the uncertainty, however, it would also include 1 when using an unbiased uncertainty estimate.

## 11. Appendix A. Data Attachment

CHIP IDENTIFIER:    K153HH

TARGET:   (T1) 30-10 7.3T

MEASURED VALUES:

The table below shows the measured critical dimension (CD) and expanded uncertainty (*k* = 2) for each feature listed. The features are designated as F1 thru F6, and this convention is maintained even when not all features have reported CDs. When the chip is oriented with the lettering up, F1 is closest to the bottom of the chip, and F6 is closest to the top. Normally, F1 is the narrowest and F6 is the widest.

**Table:**

Feature	CD (mm)	Expanded uncertainty (nm)
F1	71.0	1.4
F2	71.6	1.6
F3	115.2	1.7
F4	136.1	1.5
F5	176.0	1.2
F6	231.7	1.6
